# Evaluation of the endotracheal tube cuff pressure changes during cardiac operations under cardiopulmonary bypass

**DOI:** 10.34172/jcvtr.2023.31733

**Published:** 2023-03-16

**Authors:** Alireza Mahoori, Shahriar Khanahmadi, Shima Khanahmadi, Nasim Parvin Karami, Peyman Mokhtarzadehazar

**Affiliations:** ^1^Department of Anesthesiology, Urmia University of Medical Sciences, Urmia, Iran; ^2^Urmia University of Medical Science, Urmia, Iran

**Keywords:** Airway Management, Intubation, Endotracheal, Surgery, Cardiovascular, Cardiopulmonary Bypass

## Abstract

***Introduction:*** Tracheal intubation is used for most operations under general anesthesia. Prolonged hyperinflation of the tube cuff can compromise tracheal mucosal perfusion, and low pressure of the cuff may cause some other complications. The aim of this study was the evaluation of changes in intra-cuff pressure in patients undergoing cardiac surgeries under cardiopulmonary bypass.

***Methods:*** In an observational study 120 patient’s candidate to cardiac operations under cardiopulmonary bypass were enrolled. After induction of anesthesia and tracheal intubation by same tracheal tubes, tracheal tube cuff pressure was adjusted to 20-25 mm Hg (T0). Then the cuff pressure was measured at beginning of CPB (Cardio Pulmonary Bypass) (T1), at 30º hypothermia (T2) and after separation from CPB (T3).

***Results:*** The mean cuff pressure was 33.5±7.3, 28.9±5.4, 25.6±5.2 and 28.1±3.7 at T0, T1, T2 and T3 respectively. Intra- cuff pressure changed significantly during cardiopulmonary bypass.

***Conclusion:*** The mean intra-cuff pressure was decreased during hypothermic cardiopulmonary bypass. The decrease in cuff pressure may protect the tracheal mucosa against hypotensive ischemic injury in these patients.

## Introduction

 Tracheal intubation during anesthesia is the standard and reliable way of maintaining airways, and securing pulmonary ventilation. Cuffed endotracheal tubes are used in patients over the age of 8 years old. Endotracheal tubes are designed to maintain reliable airways. Tubes that are used in adult patients have cuffs on their distal tips, which are inflated to form a barrier with the tracheal wall to prevent pulmonary aspiration and deliver the tidal volume to the lungs. The endotracheal tube cuffs are inflated at a pressure in the range between 20 and 30 cmH_2_O, which is lower than the normal capillary perfusion pressure, a pressure below 25 mm Hg is recommended.^1,2^ Cuff inflation prevents aspiration and facilitates pulmonary ventilation in patients. A high cuff pressure also affects part of the tracheal mucus, and the pressure duration determines the progress of the damage. When the endotracheal tube cuff pressure exceeds 30 cmH_2_O for more than 15min, the capillaries are compacted, the capillary blood flow decreases, mucosal damage is caused to the trachea, and complications such as tracheal wall ischemia, throat ulcer, postoperative dysphonia, coughing, tracheal necrosis, tracheal rupture, laryngotracheal stenosis and nerve paralysis, and tracheoesophageal fistula arise. Moreover, a cuff pressure below 18 cmH_2_O increases the risk of aspiration of materials into the trachea.^3^

 The inflation of the endotracheal tube cuffs results in the formation of a barrier between the tube and the tracheal wall, there has to be no air leakage under the airway pressure required for the positive pressure ventilation, and the lungs must be protected from aspiration. Endotracheal tube cuff inflation links the cuff to the tracheal wall. It prevents the leakage of air during positive pressure ventilation and protects from aspiration. The previous endotracheal tube cuffs applied considerable pressure to the tracheal mucus and resulted in ischemia. The current endotracheal tubes are equipped with low-pressure cuffs and are designed to exert little pressure on the endotracheal tubes and minimize ischemia.^4^ The extent to which an endotracheal tube cuff should be inflated is the question asked mainly by researchers and students. In fact, a cuff should be inflated to the extent it prevents air leakage. However, the cuff pressure still varies. Preventing the over-inflation of the endotracheal tube cuffs reduces complications such as tracheal damage and, vocal cord dysfunction (due to the postoperative sore throat, and laryngeal recurrent nerve paresis).^5^ Since pilot touch is not a good indicator of cuff pressure, monitoring is suggested to be able to keep the cuff pressure in the 25-30cmH_2_O range.^4^ If the cuff is inflated correctly it prevents major aspiration while microaspiration is still possible. However, a cuff inflated under considerable pressure causes complications such as tracheal mucosal ischemia. Besides, it results in laryngotracheal stenosis, the formation of the tracheoesophageal fistula, and even severe hemorrhage.^6^ Although the cuff pressure is measured immediately following the tracheal intubation, it is a dynamic process that may be influenced by factors such as the body position, body temperature, and the mixture of the administered gases such as nitrous oxide. Some factors may also cause airway problems in patients undergoing open-heart surgery and examples include a long surgical operation, unstable hemodynamics, and pulseless blood flow during cardiopulmonary bypass surgery.^7-9^

 Surgery is often carried out using the cardiopulmonary bypass technique. In this technique, blood pumping and respiratory gas exchange are temporarily carried out by a mechanical device called an oxygenator, which is connected to the vascular system.^10^ Cardiopulmonary bypass exposes the blood to a large volume of synthetic materials, resulting in the production and secretion of chemical toxins and numerous vascular activators. The activation of the neutrophils and their subsequent presence in the pulmonary blood circulation also causes severe endothelial, epithelial, and interstitial pulmonary damage, which may be linked to an increase in the endothelial capillary permeability, a decrease in the lung capacity, and gas exchange disruption.^11^ Hence, gas exchange disruptions resulting from acute damages to the pulmonary tissues are among the common and known side effects of coronary artery bypass surgery.^12^ Despite the improvements in the cardiopulmonary bypass techniques and the intense care provided during cardiopulmonary bypass surgery, the resulting relative hypotension reduces the perfusion pressure on the tracheal mucus and may possibly damage the trachea under high cuff pressures.^13^ Considering these factors, since air density in the cuff and cuff pressure may change during cardiopulmonary bypass surgery due to the resulting hypothermia and reheating, we decide to assess these variations in a study. It is also worth noting that the endotracheal tube cuff pressure has not been monitored or measured in the heart surgeries performed in Iran. Therefore, this research was an attempt to determine whether the endotracheal tube cuff pressure changes considerably during cardiopulmonary bypass surgery and to measure its variations. Our colleagues can also study the advantages or disadvantages of correcting the cuff pressure in future research.

## Materials and Methods

 This study was conducted with the approval of the Scientific & Ethical Review Boards. After obtaining written informed consent, approximately 120 candidates for open-heart surgery were studied in a prospective observational study. Patients with signs of difficult intubation, hemodynamic instability, and candidates for emergency operations were excluded from this study. The other patients were selected using the convenience sampling technique and were anesthetized through the administration of general anesthesia. The endotracheal tube material was the same for all patients. Intubation in none of the patients was facilitated using a gel. Following the tracheal intubation (T0), the cuff pressure was measured using a cuff manometer and was set to approximately 20-25 cmH2O for all patients. During the anesthesia, the patients were connected to an anesthetic machine to undergo mechanical ventilation. The lack of leakage in the endotracheal tube cuff was also ensured. All patients were in the same condition during the cardiopulmonary bypass. Thereafter, the cuff pressure was measured and recorded at times T1 (the beginning of cardiopulmonary bypass), T2 (the 30-degree hypothermia period), and T3 (after separation from the CPB). In this process, a cuff was placed next to the endotracheal tube with one end of the cuff connected to the manometer. The cuff pressure was measured and recorded by pressing a button when needed. The patients were never detached from the ventilator to measure the cuff pressure and were not deprived of the routine anesthetic and surgical processes. Furthermore, routine cuff pressure has never been monitored continuously and precisely o in the surgeries performed in Iran, the necessity of correcting the cuff pressure has never been mentioned in any reference, and the cuff pressure was measured only three times in this study. Finally, the collected data was statistically analyzed in SPSS 18 using the paired samples statistics.

 It is worth restating that determining whether considerable changes occur in the endotracheal tube cuff pressure during cardiopulmonary bypass and measuring these changes were the overarching goals of this study. Other studies can be conducted in the future based on the findings from this research to explore the advantages or disadvantages of correcting the cuff pressure. However, explaining whether pressure correction benefits or harms the patients is beyond the scope of this research.

## Results

 The mean age of the patients was 54.35 ± 10.42 years (a minimum of 40, a maximum of 68 years, and a median of 56 years), 69 (57.5%) and 51 (42.5%) of the study patients were male and female, respectively (Table 1).

**Table 1:** Distribution of absolute and relative frequency of sex in the studied population

**Sex**	**Absolute frequency**	**Relative frequency**
Men	69	57.5
Women	51	42.5
Total	40	100

 The base initial ETT (endotracheal tube) cuff pressure was 33.55 ± 7.33 (cm H2O) (a minimum of 27, a maximum of 58, and a median of 30mm Hg) and ETT cuff pressure at the beginning of the cardiopulmonary bypass was also 28.95 ± 5.45 (cm H2O) (minimum: 18, maximum: 44, and median: 28 mm Hg). According to the results, the mean difference between the initial cuff pressure and the cuff pressure at the beginning of the cardiopulmonary bypass was 4.65 ± 3.54 (cm H2O). Moreover, according to the paired samples statistics, there was a significant difference between the initial ETT cuff pressure and the endotracheal tube cuff pressure at the beginning of the cardiopulmonary bypass (*P* < 0.001).

 The ETT cuff pressure at the time of 30-degree hypothermia was 25.65 ± 5.23(mm Hg) (minimum: 14, maximum: 38, and median: 26 mm Hg). The results suggest that the mean difference between the initial ETT cuff pressure and the endotracheal tube cuff pressure at the time of cooling was 7.90 ± 4.69 (cmH2O). The paired samples statistics also revealed a significant difference between the initial endotracheal tube cuff pressure and the endotracheal tube cuff pressure at the time of cooling (*P* < 0.001).

 The mean initial ETT cuff pressure was 33.55 ± 7.33(cmH2O) (minimum: 27, maximum: 58, median: 30mm Hg), and the mean ETT cuff pressure following termination of CPB was 28.10 ± 3.78(mm Hg) (minimum: 22, maximum: 40, and median: 28mm Hg). According to the research findings, the mean difference between the initial endotracheal cuff pressure and the endotracheal tube cuff pressure after detachment from the pump was 5.45 ± 4.67(cmH2O).The paired samples statistics reflected the significant difference between the initial endotracheal tube cuff pressure and the ETT cuff pressure following detachment from the pump (*P* < 0.001). (Table 2)

**Table 2:** Tracheal cuff pressure (mean ± SD) at (T0, T1, T2, T3) in the studied population

**Time Intervals**	**Cuff Pressure (cm2o)** **Mean ** ± ** SD**	**Comparing the variable**		***P*** ** value**
After Tracheal Intubation (T0)	33.55 ± 7.33	Initial ETT cuff pressure(T0)	(T1)	0.001
Cardiopulmonary bypass Starting time (T1)	28.95 ± 5.45		(T2)	0.001
At 30º hypothermia (T2)	25.65 ± 5.23		(T3)	0.001
After separation from CPB (T3)	28.10 ± 3.78			

**Figure 1:**
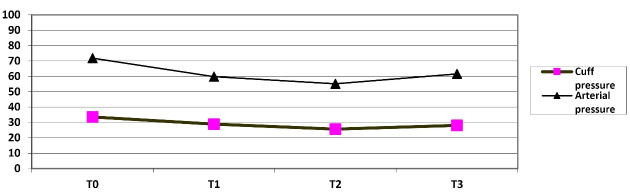
Mean arterial pressure and cuff pressure at (T0, T1, T2, T3) in the studied population

 The mean arterial pressure (MAP) at the time T0 was 71.85 ± 9.22cmH2O, at the beginning of cardiopulmonary bypass (T1) was 59.80 ± 8.08 mm Hg, at 30º hypothermia was 55.15 ± 10 cmH2O, and after separation from the CPB was also 61.60 ± 9.53 mm Hg (Tables 3, 4 and Figure 1).

**Table 3:** Mean arterial pressure (mean ± SD) in the studied population

**Time Intervals**	**Mean arterial pressure (mm Hg)** **(Mean ** ± ** SD)**	**Minimum**	**Maximum**	**Median**
After Tracheal Intubation (T0)	71.85 ± 9.22	50	87	74
Cardiopulmonary bypass Starting time (T1)	59.80 ± 8.08	42	79	58.5
At 30º hypothermia (T2)	55.15 ± 10.00	40	77	52.5
After separation from CPB (T3)	61.60 ± 9.53	47	82	60

**Table 4:** Tracheal cuff pressure and Mean arterial pressure (mean ± SD) in the studied population

**Time Intervals**	**Cuff pressure (cmH2O)** **Mean and SD**	**Mean arterial pressure (mm Hg)** **Mean and SD**
After Tracheal Intubation (T0)	33.55 ± 7.33	71.85 ± 9.22
Cardiopulmonary bypass Starting time (T1)	28.95 ± 5.45	59.80 ± 8.80
At 30º hypothermia (T2)	25.65 ± 5.23	55.15 ± 10.00
After separation from CPB (T3)	28.10 ± 3.78	61.60 ± 9.53

## Discussion

 The endotracheal tube cuff pressure and the over-inflation pressure are important during long surgical procedures. Despite the risk of over-inflation at the beginning of cuff inflation, it should be noted that the endotracheal tube cuff pressure is a dynamic process determined by different factors.^14,15^ Wujtewicz MA et al reported that in most of the patients exposed to general anesthesia and tracheal intubation through direct laryngoscopy, the tracheal tube cuffs were over-inflated at the end of the general anesthesia administered by anesthesiologists.^16^ Sole et al concluded that the cuff pressure was in the normal 20-30cmH2O range only in 54% of the patients. Besides, the cuff pressure level was high and low in 16% and 30% of the patients, respectively.^17^ These findings are indicative of the lack of control over the endotracheal tube cuff pressure in hospitalized patients or the incorrect inflation of the endotracheal tube cuffs. In various studies, the pressure in the endotracheal tube cuffs was measured in long surgical operations and it was found out that the cuff pressure necessitates constant monitoring as it may increase or decrease during surgical procedures.^18^ Our research also confirms this finding. As mentioned, the excessive the endotracheal tube cuff pressure may impair the tracheal wall mucosal perfusion, resulting in postoperative complications. These complications may include sore throat, dysphonia, subglottic complications, tracheal rupture, laryngotracheal stenosis, and laryngeal nerve paresis.

 The results of this study mirrored the positive correlation between the temperature and the variations of the endotracheal tube cuff pressure, which is in line with the findings reported by Neto et al^19^ and Indada et al.^20^ Moreover, Neto et al analyzed the effect of temperature on the endotracheal cuff pressure during cardiopulmonary bypass surgeries in France and stated that high temperature increases the endotracheal tube cuff pressure. In this regard, Indada et al stated that the cuff pressure during cardiopulmonary bypass decreases to 8cmH2O prior to heating and rises to 17cmH2O after heating, i.e. there is a direct relationship between the temperature variations and the cuff pressure.

 Kako et al analyzed the ETT cuff pressure during congenital heart disease surgeries performed using the cardiopulmonary bypass technique and measured cuff pressure at different times and temperatures. They indicated that the cuff pressure decreases with body temperature,^18^ which complies with our findings regarding cardiopulmonary bypass surgeries on adult patients. The decrease in temperature reduces the tracheal tube cuff pressure, which can control the effect of the decrease in the systemic pressure and the pulseless flow of blood during cardiopulmonary bypass that affects tracheal mucosal perfusion. In other words, a decrease in cuff pressure protects the tracheal mucus at the time of hypotension during surgeries performed under cardiopulmonary bypass. The importance of controlling the endotracheal tube (ETT) cuff pressure is also stressed in other studies.^21,22^

 Even under a normal cuff pressure, the resulting hypotension and the decreased tracheal mucosal perfusion can inflict damage on the trachea. However, this does not occur during heart surgeries because the drop in blood pressure during cardiopulmonary bypass is in line with the decrease in the ETT cuff pressure due to hypothermia and prevents any damage and this finding is reflected in the results of this study. The study by Grant T. indicated that the ETT cuff pressure grows slightly with an increase in the ambient temperature, which may be substantially important in patients suffering from hyperthermia or hypothermia as it may change the cuff pressure and result in the side effects of the increase or decrease in the cuff pressure.^23^

 According to our observations, the ETT cuff pressure decreased at the beginning of the cardiopulmonary bypass and prior to cooling. This could be attributed to the fact that the pressure in the low-pressure large-volume cuffed tubes is determined by the pressure acting on the tracheal wall, which is determined by the thorax pressure and the pleural pressure (Ppl). When the heart and lungs are bypassed and the mechanical ventilation of the lungs stops, the aforesaid pressure decreases, reducing the cuff pressure. In other words, regardless of hypothermia, which definitely contributes to the decrease in the cuff pressure, the cardiopulmonary bypass process lowers the ETT cuff pressure in itself.

 In some studies, it is stated that nitrous oxide increases ETT cuff pressure after flowing into the ETT cuff.^20^ The effect of the other gases on the ETT cuff pressure has also been the subject of other studies. In the two studies by Ishigoro et al^24^ and Miura and Suzoki,^25^ it is reported that the nitrous oxide and xenon gases travelling through the endotracheal tube pass through the tube membrane and penetrate the cuff, thereby increasing the cuff pressure. The research findings also imply that a gas exchange occurs due to the pressure gradient difference between the gases around the cuff (passing through the tube) and the gases in the cuff, changing the ETT cuff pressure. Hence, nitrous oxide is often not used in these patients during heart surgeries to avoid an increase in the size of air bubbles in the course of heating.

 Specialists are, therefore, recommended to ensure the adequate management of the ETT cuff pressure in patients undergoing mechanical ventilation. Numerous studies have also suggested that the ETT cuff pressure is variable and decreases over time, and thus patients can be protected from the consequences of the cuff pressure fluctuations by regularly monitoring cuff pressure using precise tools.

## Conclusion

 Based on our findings it is concluded that the endotracheal tube cuff pressure decreases significantly when cardiopulmonary bypass starts and the decrease continues into hypothermia. After the patient is heated and detached from the cardiopulmonary pump, the cuff pressure rises again, yet it never reaches the initial pressure. Meanwhile, the average MAP (mean arterial pressure) follows the same descending trend in the course of bypass, and since these variations are in line, no damage is caused to the tracheal mucus despite the long duration of the surgical operation and the long intubation process.

## Acknowledgements

 Clinical Research Development Unit of Imam Khomeini Hospital, Urmia University of Medical Sciences, Urmia, Iran

## Competing Interests

 No conflict of interests was declared

## Ethical Approval

 All authors disclose that informed consent was obtained and the manuscript has the approval of ethics committee. However, since this is a prospective observational study and not a clinical trial, no intervention was performed on the patient

## Funding

 Vice Chancellor for Research of Urmia University of Medical Sciences and Azad University of Tabriz.
